# A Manual Segmentation Tool for Three-Dimensional Neuron Datasets

**DOI:** 10.3389/fninf.2017.00036

**Published:** 2017-05-31

**Authors:** Chiara Magliaro, Alejandro L. Callara, Nicola Vanello, Arti Ahluwalia

**Affiliations:** ^1^Centro di Ricerca “E. Piaggio”, Università di PisaPisa, Italy; ^2^Dipartimento di Ingegneria dell'Informazione, Università di PisaPisa, Italy

**Keywords:** manual segmentation, confocal stacks, CLARITY, neurons, L7GFP, segmentation goodness

## Abstract

To date, automated or semi-automated software and algorithms for segmentation of neurons from three-dimensional imaging datasets have had limited success. The gold standard for neural segmentation is considered to be the manual isolation performed by an expert. To facilitate the manual isolation of complex objects from image stacks, such as neurons in their native arrangement within the brain, a new Manual Segmentation Tool (ManSegTool) has been developed. ManSegTool allows user to load an image stack, scroll down the images and to manually draw the structures of interest stack-by-stack. Users can eliminate unwanted regions or split structures (i.e., branches from different neurons that are too close each other, but, to the experienced eye, clearly belong to a unique cell), to view the object in 3D and save the results obtained. The tool can be used for testing the performance of a single-neuron segmentation algorithm or to extract complex objects, where the available automated methods still fail. Here we describe the software's main features and then show an example of how ManSegTool can be used to segment neuron images acquired using a confocal microscope. In particular, expert neuroscientists were asked to segment different neurons from which morphometric variables were subsequently extracted as a benchmark for precision. In addition, a literature-defined index for evaluating the goodness of segmentation was used as a benchmark for accuracy. Neocortical layer axons from a DIADEM challenge dataset were also segmented with ManSegTool and compared with the manual “gold-standard” generated for the competition.

## Introduction

Understanding how the brain works is arguably one of the greatest challenges of our time (Alivisatos et al., 2012). A fundamental limitation for exploring the function of complex neural circuits and their alterations in pathological brain processes is our lack of knowledge on the microarchitecture and organization of neurons in the brain. To understand the structure-function relationship in the brain, the first step is to identify the 3D (three-dimensional) arrangement of a single cell in its native environment within the brain from neuroimaging data. This key task could enable studying the morphological properties of neurons, to investigate the factors influencing neural development and alterations related to specific diseases (Iannicola et al., 2000; Solis et al., 2007; Billeci et al., 2013), the relationships between neuronal shape and function (Costa Lda et al., 2002; Brown et al., 2005; White, 2007) or the effects of specific compounds on neuron geometry that could give useful information for designing new drugs (Seeman and Lee, 1975; Robinson and Kolb, 1999). In addition, single cell reconstructions can be used to simulate electrophysiological behavior based on empirical microscopic data (Hines and Carnevale, 1997), to generate models that can be used to make predictions about high-level organization (Egger et al., 2014), or to develop algorithmic descriptions of neurons based on statistical analyses (Ascoli and Krichmar, 2000; Ascoli, 2002).

Numerous advances in imaging have enabled inspection of the brain at cellular resolution. In particular, confocal and multi-photon microscopy have revolutionized neuro-biological discoveries by allowing the study of micro-structure (Ntziachristos, 2010). However, even these methods cannot image intact brain samples more than few hundred micrometers in thickness (Oheim et al., 2001). To overcome this limit, the recently developed clarification methods render the brain optically transparent (Regehr et al., 2009; Hama et al., 2011; Chung and Deisseroth, 2013; Chung et al., 2013; Kuwajima et al., 2013; Ertürk et al., 2014; Poguzhelskaya et al., 2014; Richardson and Lichtman, 2015; Magliaro et al., 2016), allowing a notable increase of light penetration depth, which enables the visualization of the global arrangement of large brain cell populations.

While brain tissue imaging methods are constantly improving in their performance, powerful computational algorithms and tools to segment neural structures, such as soma and neurites, are still lacking (Meijering, 2010), despite the organization of competitions in the field [e.g., the DIADEM (Digital Reconstruction of Axonal and Dendritic Morphology) challenge (Ascoli, 2009)]. In fact, different tools for the extraction of 3D neural structures have been presented in the literature (Kass et al., 1988; Osher and Sethian, 1988; Glaser and Glaser, 1990; Paragios et al., 2001; Dima et al., 2002; Vese and Chan, 2002; Rohlfing et al., 2004; Wolf et al., 2009; Basu et al., 2013; Mukherjee et al., 2013; Quan et al., 2016), but none of them represents a robust system with general applicability. Beyond the technical limitations of these approaches, two basic aspects have to be underlined: on one hand, since these tools usually just perform three-dimensional neuron tracing through skeletonization, the neurons extracted have scarce information on their morphology (i.e., surface and volume of the whole neuron, dendrite diameters). On the other hand, the methods for validation of the three-dimensional reconstruction are often difficult and arbitrary. To date, manual segmentation operated by neuroscience experts is considered the gold standard (Al-Kofahi et al., 2003; Meijering, 2010) and is considered necessary as a yardstick against which new reconstruction algorithms can be compared.

During the DIADEM challenge, a metric was established to evaluate the goodness of segmentation. This metric, freely released open-source on the DIADEM challenge website, quantifies the similarity between two different reconstructions of the same neuron (i.e., manual and via the algorithm being evaluated) by matching the location of the bifurcations and terminations between the reconstructed arbors. This method was purposely designed to capture the most critical aspects in neuronal segmentation. However, new methods continue to be validated in different ways in different reports. How the manual segmentation is operated remains elusive and most of the evaluations are not duly detailed, making it difficult to perform comparative assessments of the goodness of reconstruction.

The most popular tool for manual segmentation from three-dimensional datasets is represented by Neurolucida (MicroBrightField, Inc., Williston, VT; Glaser and Glaser, 1990). Despite the power of the software and its packages, it is very expensive and cannot always be justified as a sustainable investment, particularly for the validation of novel algorithms. A good alternative to Neurolucida is the ITK-based 3D Slicer (Fedorov et al., 2012); however its use is confined to trained experts. Indeed, the interface is not intuitive and manual segmentation is quite laborious.

Other tools such as FilamentTracer (Imaris Bitplane Inc., Zurich), only provide methods for manual neuron tracing—and not segmentation. On the other hand, software like ITK-SNAP (Yushkevich et al., 2006) or CellSegm (Hodneland et al., 2013) are not purposely developed for manual segmentation of three-dimensional structures: this makes the operation tricky and labor-intensive. Finally, some tools have been developed for computer-assisted segmentation (for example the Segmentation Editor ImageJ plug-in Schindelin et al., 2012 or the method developed by Schmitt et al., 2004), but, since they rely on internal criteria to “help” segmenting, they cannot be considered good candidates for validating other tools. To the best of our knowledge, none of the above returns information on the time spent to perform the segmentation, which can be useful, for example, to compare the outputs of different tools and objectively judge their performance.

To address these limitations, we implemented a user-friendly tool, ManSegTool (Manual Segmentation Tool) to facilitate the manual segmentation of neurons from image stacks. Firstly, ManSegTool can be a valid alternative for neuron segmentation when the automatic tools available in the state of art have limited success for a specific application. Moreover, it could also be considered as robust and easy-to-use manual segmentation tool for the validation of neuron reconstruction software, comparing the outputs in terms of computational timing using goodness of segmentation indices defined in the literature (Zhang, 1996).

In this paper, after outlining the features of ManSegTool, we describe how the tool was rigorously assessed for accuracy and precision, by segmenting Purkinje cells from 3D image stacks of clarified murine cerebellum acquired using a confocal microscope. In order to show that ManSegTool can deal with different neuron types, an online dataset representing Neocortical Layer neurons from the DIADEM challenge was also manually segmented and compared to the manual segmentation available online. The comparison was quantified using a metric similar to the one employed in the challenge.

## An outline of ManSegTool

**Figure 1:**
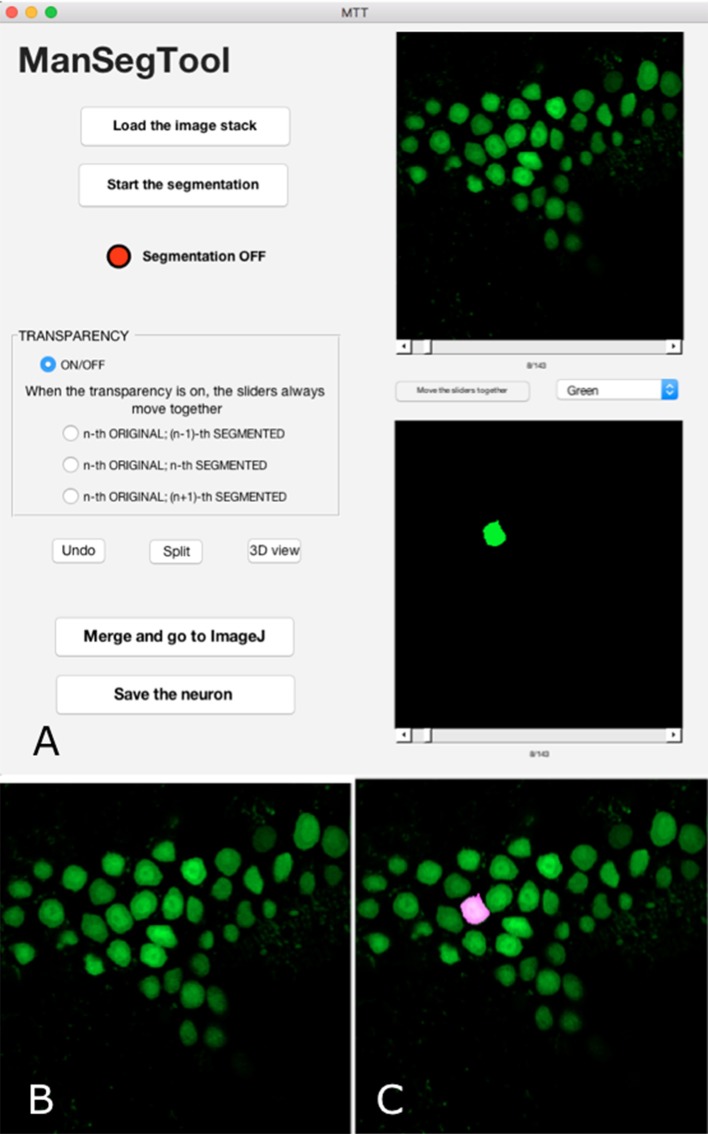
**(A)** ManSegTool Graphical User Interface. **(B)** An example of the i-th image from a confocal stack automatically shown to zoom and segment the object of interest (i.e., neurons). **(C)** An example of the transparent image overlapping which helps to keep track of the segmented object between the stack planes.

ManSegTool is an open-source software, developed in Matlab® (The Mathworks-Inc, USA) and downloadable at https://mansegtool.wordpress.com/. It has a Graphical User Interface, shown in Figure 1A, designed to facilitate the manual segmentation of complex objects (e.g., Purkinje neurons in mouse cerebella) from three-dimensional stacks (which may be acquired with a confocal microscope). Users can load a stack, segment one or more three-dimensional objects within it, while keeping track of the time spent to perform each segmentation and monitoring the accuracy of the manually selected regions. They can also view and save the results obtained. A detailed description of all the features of the software is reported in the following sections.

### Original stack loading and datamatrix construction

The user can load an 8-, 12-, or 16-bit grayscale ^*^.tiff image stack, that is converted into a three-dimensional matrix M^*^N^*^Z (i.e., Z images of size M^*^N stacked one on top of another) matrix. All Z images can be displayed in the upper panel of the GUI by scrolling a slider.

The GUI automatically creates a structure, nominated “datamatrix” and stores one or more segmented neurons and their related information extracted during the processing. For each segmented object, the datamatrix is purposely designed to provide and store:

- The volumetric representation as an M^*^N^*^Z matrix of logical values, that are true (i.e., 1) for the foreground and false (i.e., 0) for the background.
- The information needed to define the shape of the object surface (in terms of faces and vertices constituting the polygon mesh) from the volumetric binary data:
- The time (in seconds) spent to perform each segmentation.

Alternatively, the user can load a previously saved datamatrix, where segmented structures and the related information have already been stored. After that, he/she can choose to start a new segmentation *ex-novo*, or continue the processing on a partially segmented neuron already stored in the file.

The result of the manual segmentation is shown in the lower panel of the GUI (Figure 1A). The toggle button between the panels allows the user to scroll the images together: this may be useful to facilitate the operation and avoid errors.

### Starting segmentation

After choosing the i-th image (with i = 1:Z) of the original stack, the operation can proceed using a designated button: a figure is automatically shown (Figure 1B), representing the i-th image, and the red LED on the GUI switches to green. In general, the counter measuring the time spent to perform the segmentation only runs when the LED is green and stops when it is red.

The user can iteratively zoom the i-th image to magnify a region of interest and then, by clicking and dragging the mouse, draw a freehand shape representing the section of the complex three-dimensional object to be segmented. If the shape the user draws is not perfectly closed, the algorithm automatically connects the last point drawn with the first one. It is possible to draw more than one region, to include neuron branches. Finally, the algorithm returns a binary image that is the same size of the i-th image with ones inside the freehand selection and zeros elsewhere. The i-th position of the volumetric binary dataset is automatically displayed in the lower panel of the GUI. To continue the segmentation on a consecutive image (i.e., the i−1-th or the i+1-th one), the user scrolls the slider and the figure is automatically refreshed, ready to be segmented.

If the user is not satisfied with the freehand selection or wrongly chooses a region not belonging to the object to segment, the “Undo” button deletes these unwanted parts after clicking on them. In case of optical artifacts in the confocal datasets, some regions of the image may be ambiguous. For example, neurites too close each other can appear as the same structure, although they belong to different branches, of the same neuron or to a different ones. The “Split” button allows the user to manually separate the structures by simply drawing a line between them.

Sometimes the segmentation can be particularly tricky, for example in case of a slice containing many neuron branches to be followed in different images constituting the original stack. In order to facilitate the manual segmentation processing, the GUI provides transparent overlapping of the i-th original image with the i−1-th or the i+1-th binary one (Figure 1C). The transparency allows the user to easily segment the objects because it helps to keep track of structures in consecutive parts of the entire volume.

### Graphic display and datamatrix saving

At any time, the user can pause the segmentation to display the three-dimensional object as a polygonal mesh and monitor the results.

In addition, one can click on the “Merge and go to ImageJ” button to create and save a new stack in which the segmented object is highlighted within the original stack. The visualization of this dataset can be performed using the 3D Viewer ImageJ plug-in (Rasband, 2011) or other software with a 3D viewer tool for grayscale three-dimensional stacks.

At the end of the operations, the datamatrix can be automatically saved in a user-specified folder, and, if needed, re-loaded to start segmenting another neuron in the same confocal dataset or to continue a previously started one.

**Figure 2:**
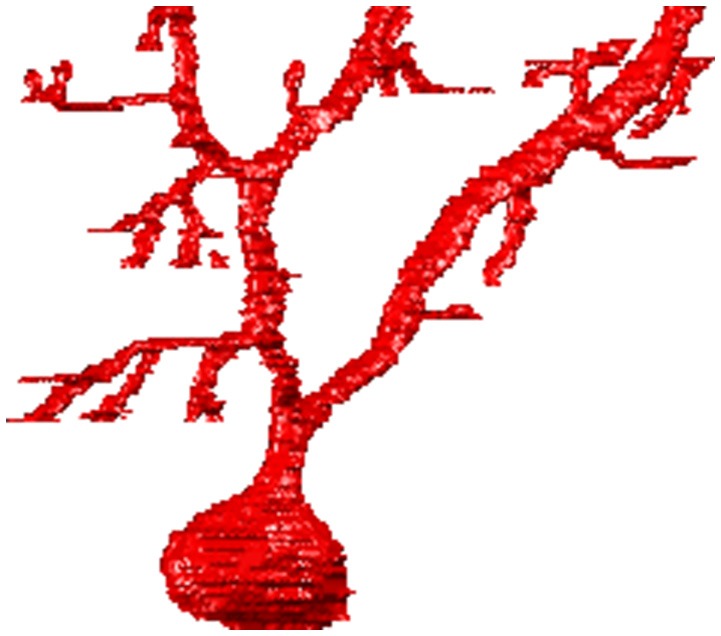
An example of the 3D graphical output of the ManSegTool, representing a Purkinje cell from mouse cerebellum manually segmented from a confocal stack.

A typical example of the graphical output of the ManSegTool at the end of the segmentation (i.e., the single segmented neurons) is shown in Figure 2, while Video 1 (see Supplementary Materials) is an animation of the result of the “Merge and go to ImageJ” function.

## Materials and methods

### PCs segmentation

#### Murine tissue preparation brain clarification

A L7GFP mouse was obtained from the Department of Translational Research New Technologies in Medicine and Surgery of the University of Pisa (Italy). The experiments were conducted in conformity with the European Communities Council Directive of 24 November 1986 (86/609/EEC and 2010/63/UE) and in agreement with the Italian DM26/14. Experiments were approved by the Italian Ministry of Health and Ethical Committee of the University of Pisa. The mouse was processed as in Magliaro et al. (2016). Briefly, the adult mouse was anesthetized and perfused with 20 mL of ice cold Phosphate Buffered Saline (PBS 1X, Sigma-Aldrich, Milan, Italy) and then with 20 mL of ice cold hydrogel solution (4% acrylamide, 0.05% bis-acrylamide (BioradLabInc., California, USA), 4% formaldehyde (PFA, Sigma-Aldrich) and 0.25% VA-044 thermally triggered initiator (Wako Chemicals, Neuss, Germany). After brain extraction and hydrogel polymerization, 1 mm-thick slices were cut using a Leica VT1200s vibratome (Leica Microsystems, Nussloch, Germany), obtaining two cerebellum slices. The slices were immersed in 20 mL of clearing solution (200 mM Boric Acid (Farmitalia Carlo Erba spa, Italy) and 4% Sodium Dodecyl Sulfate (SDS, Sigma-Aldrich), pH adjusted to 8.5 by adding 1M NaOH) for 5 days. As demonstrated in Magliaro et al. (2016), this length of time for brain clarification is the best compromise between the increase in light penetration and the decrease in fluorescent signal.

#### Confocal stack acquisition

After the clarification step, slices were mounted on a glass slide with FocusClear™ (Celexplorer Labs Co., Hsinchu, Taiwan) and then acquired using a confocal microscope (Nikon A1). In particular, acquisitions were performed using a 40x objective with a pixel-to-micron ratio of 0.62 μm/pixel on a 512 × 512 matrix. During the acquisition, no limits were imposed on the depth of the dataset, thus the height of different z-stacks is variable for each acquisition and dependent on biological variability (i.e., the different spatial distribution of PCs in different mouse cerebella). In order to minimize signal loss during the acquisitions (i.e., due to fluorescence bleaching), the z-step size selected was double that of the in-plane resolution.

#### Manual segmentation using ManSegTool

All the confocal acquisitions were processed with the ManSegTool in order to segment the PCs. In particular, 6 expert neuroscientists with no specific computational skills from the School of Medicine, University of Pisa, segmented the same 4 PCs from 4 different confocal stacks. The neuroscientists worked on their own on the segmentation, so they did not influence each other during the operation.

#### ManSegTool performance evaluation

In order to evaluate the performance of ManSegTool, morphometric parameters were extracted from the segmented neurons. In particular, the volume and the area obtained from the isosurface data were extracted for all the neurons segmented by the users.

Since both the volume and the area give global information about the precision of segmentation, the Sholl analysis (Sholl, 1955) on the neuron skeleton (Lee et al., 1994) was also performed, to locally evaluate the complex arborization of the dendritic tree. In particular, this method evaluates neurite arborization by drawing a series of concentric spheres of increasing radii around the cell soma and then counting the number of times the neurites intersect with each sphere. The Sholl analysis was performed using the ImageJ (Rasband, 2011) plug-in described in Ferreira et al. (2014) and downloadable at https://imagej.net/Sholl_Analysis.

In addition, a further analysis to evaluate the goodness of neuron segmentation was also performed. In particular, the Gray-level Uniformity index (GU) was calculated, since it does not require *a priori* knowledge of a reference segmentation (Zhang, 1996).

#### Statistical analysis

To evaluate user differences in segmenting the four objects, the Friedman test was carried out on neuron volume, neuron area and GU respectively, setting significance at *p* < 0.05.

As regards the Sholl analysis, the Friedman test was performed for each neuron segmented by the 6 users, comparing the number of neurite intersections with the spheres of increasing radii, again setting significance at *p* < 0.05.

### DIADEM challenge dataset segmentation

To show that ManSegTool is able to segment different neuron types, a dataset from the DIADEM challenge was also manually segmented. In particular, a two-photon laser scanning microscopy dataset representing Neocortical Layer 1 Axons was downloaded from http://diademchallenge.org/neocortical_layer_1_axons_readme.html. Then, the six neuroscience experts segmented the same neuron with the ManSegTool.

#### ManSegTool performance evaluation

Since a corresponding gold standard for manually-traced digital reconstructions, obtained with Neurolucida (Glaser and Glaser, 1990), is also available for the DIADEM dataset, we evaluated ManSegTool's performance using a metric similar to the one designed during the DIADEM challenge. The DIADEM challenge metric is a topology-based multi-step process that scores the connection between each node in the gold standard reconstruction based on whether or not the test reconstruction captures that connection (Gillette et al., 2011). In particular, the comparison is performed on a reference set of points of interest within the dataset (i.e., three-dimensional coordinates of bifurcations, branches, end-points, etc.). Specifically, the data are stored in a ^*^.swc file and evaluated through the application of an Euclidean distance threshold, that involves separate checks for XY distance and Z distance, as the on-plane and the intra-plane resolutions are often different.

Since the ManSegTool output is a structure constituting the whole neuron, and not only a set of points of interest detected by the user, we searched the ManSegTool output points which minimized the 3D-Euclidean distance from the ^*^.swc file points through an *ad-hoc* developed Matlab (The Mathworks Inc.) script. In this way it was possible to obtain a one-to-one mapping between the DIADEM reference points of interest and the ManSegTool ones. Then, for each pair of points, the XY and Z Euclidean distance were calculated. The points whose distances were bigger than the threshold value defined for the specific dataset in the DIADEM challenge metric were marked as “missing”; on the other hand, those points whose distance was below the threshold were marked as “matched points”.

## Results

### PCs performance evaluation

**Figure 3:**
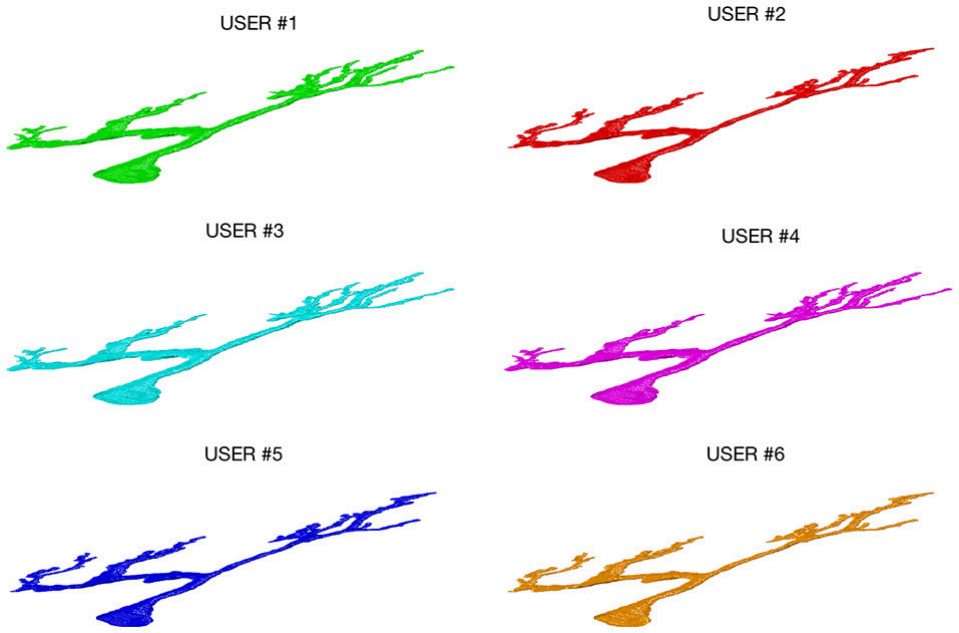
A Purkinje cell from a clarified mouse cerebellum slice acquired using a Nikon1 confocal microscope and segmented by the six users using the ManSegTool.

Figure 3 shows an example of a neuron extracted by each of the 6 experts: all the users were able to entirely follow neurite arborization, without missing segmentation of any part of the neuron within the confocal stack.

**Figure 4:**
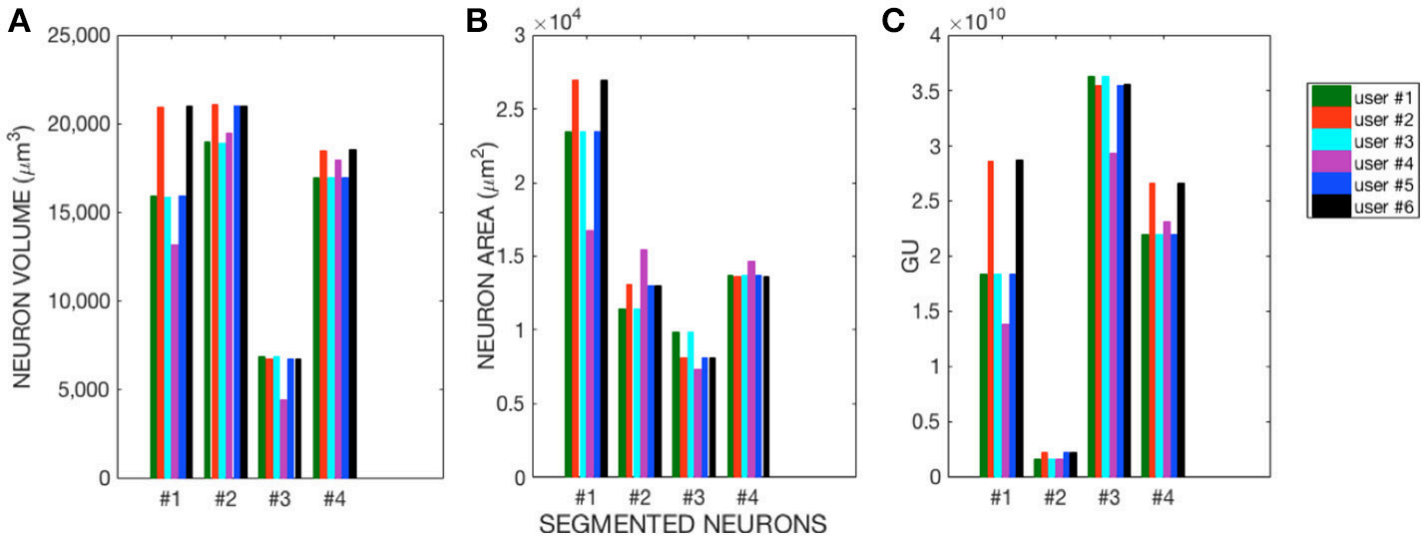
**(A)** Neuron Volume (*p* = 0.223), **(B)** Neuron Area (*p* = 0.965), and **(C)** GU index (*p* = 0.182) for the four Purkinje cells segmented by each of the six users.

**Figure 5:**
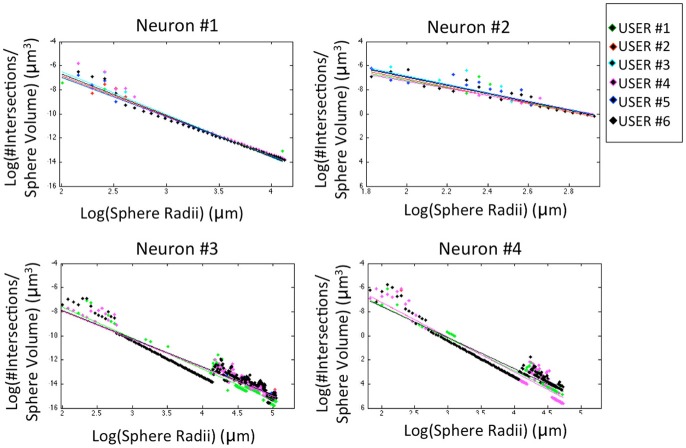
Sholl analysis for the four neurons segmented by the six experts. For a better visual comparison of the results, data for each user are fitted to a straight line (*p*-values: Neuron #1 = 0.058; Neuron #2 = 0.235; Neuron #3 = 0.066; Neuron #4 = 0.137).

The morphological features and the homogeneity index are shown in Figure 4, while the Sholl analysis to evaluate local changes in the neurite distribution is reported in Figure 5. The figures represent the log-log ratio of the number of intersections in the Sholl sphere volume as a function of Sholl sphere radius. The Friedman test confirms that there are no statistically relevant differences between the observers in terms of neuron volume (*p*-value: 0.223), neuron area (*p*-value: 0.965), GU (*p*-value: 0.182) and number of neurite intersections with the spheres (*p*-values: 0.058, 0.235, 0.066, 0.137).

### DIADEM challenge dataset evaluation

**Figure 6:**
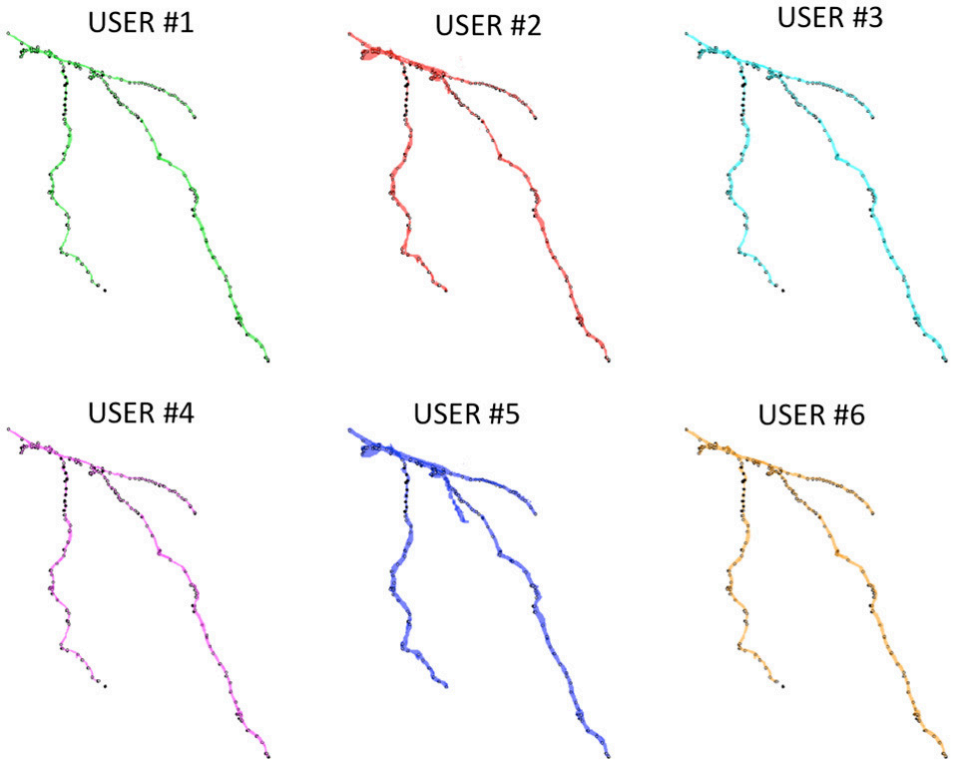
The 3D graphical output of the ManSegTool tool, representing Neocortical Layer 1 axons from mouse brain manually segmented by six neuroscientists. On each neuron, the white marker are the DIADEM reference points of interest, while the black ones are those extracted from the segmented neuron (as detailed in Section ManSegTool Performance Evaluation).

Figure 6 shows the Neocortical Layer 1 axon from the DIADEM challenge dataset segmented by the six users. On each neuron, both the three-dimensional coordinates extracted from the gold standard ^*^.swc (i.e., the white markers in Figure 6) and those extracted from the segmented neuron (i.e., the black markers in Figure 6) are also plotted. As shown in the figure, all the users were able to precisely follow the axon arborization. Note that the DIADEM metric cannot be exhaustive for neuron segmentation, since it just gives geometrical, skeleton-based information about neuron shape, while ManSegTool allows volume segmentation.

To quantify the goodness of the segmentation with a metric similar to the one defined during the DIADEM challenge, we report the percentage of matched points on both XY plane and Z axis for all the six neuroscientists. In particular, the three-dimensional coordinates for which the Euclidean distances are below the thresholds (i.e., 4.76 pixels for the XY plane and 17 pixels for the Z axis) are considered “matched points.” As shown in Table 1, all the users were able to accurately follow axon arborization using ManSegTool: in fact, at least the 95.46% of the points are considered “matched points” on the XY plane, while 99.36% of the points are considered “matched points” on the Z one.

**Table 1:** ManSegTool scoring in the DIADEM challenge for a neocortical layer 1 axon.

**User**	**XY matched points (%)**	**Z matched points (%)**
#1	98.06	99.36
#2	96.11	99.36
#3	96.11	99.36
#4	95.46	99.36
#5	97.41	99.36
#6	97.41	100

*The percentage of pairs of points obtained by ManSegTool within 4.76 pixels (XY) or 17 pixels (Z) of a corresponding reference marked pair by the DIADEM challenge*.

**Figure 7:**
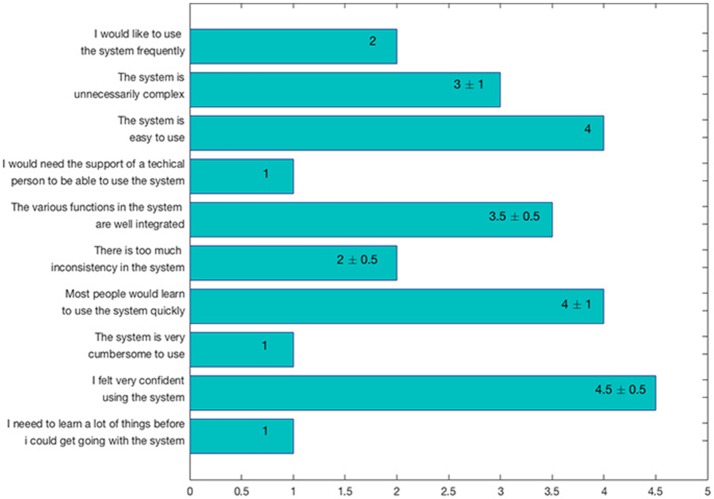
SUS questionnaire results, expressed as median ± MAD.

Users were also asked to answer to the Scale Usability Test (SUS) questionnaire (Brooke, 1996), a reliable and frequently used tool for measuring GUI usability. In particular, the SUS consists of 10 items with five response options (i.e., from strongly agree to strongly disagree), used to evaluate ManSegTool's usability. The results, reported in Figure 7 as median ± median absolute deviation (MAD), show that the tool is easy to use and learn, since it does not require specific training.

## Discussion

The reconstruction and the study of neuronal morphology from three-dimensional image stacks is considered a crucial task in neuro-scientific research, as it could help elucidate the relationship between structure and function in the brain. Despite the considerable efforts channeled in this field, the task is far from being solved: in fact, an automatic, general-purpose and robust method to deal with the large variability of neuro-image datasets is still lacking.

ManSegTool is an open-source software purposely developed to facilitate the manual segmentation of complex objects in a three-dimensional environment represented by image stacks. In particular, the software is constructed in a GUI framework written in Matlab that allows the user to scroll down the images constituting the stack and to manually identify the structures of interest constituting the object to segment. In this work, we show that ManSegTool is a precise and accurate tool for manual segmentation by evaluating morphometric parameters and a homogeneity index to measure respectively the accuracy of segmentation and the goodness of the operation.

**Figure 8:**
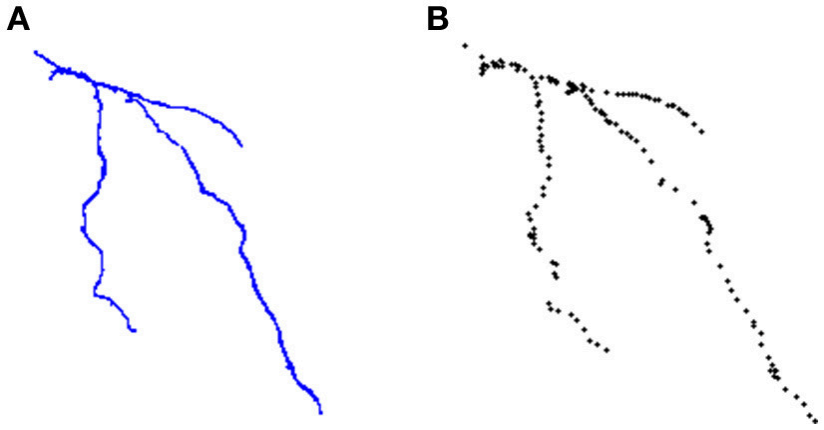
**(A)** An example of neuron segmented by a neuroscientist from the DIADEM challenge two-photon microscopy dataset. **(B)** The set of points of interest stored in the ^*^.swc file for that neuron.

ManSegTool was also tested on different types of datasets (i.e., confocal stacks of Purkinje cells from clarified mouse cerebella and Neocortical Layer 1 Axons from two-photon microscopy datasets from the DIADEM challenge). The positive results obtained are a strong indication of the general applicability of ManSegTool to manually segment any kind of datasets in order to isolate a single neuron. Moreover, as shown in Figure 8, additional data for the definition of a gold standard in neuron segmentation can be obtained, such as neuron surface and volume, which increases the information derived from the segmentation process. We show that ManSegTool is easy to use for non-expert users. Notably, as illustrated in Figure 7, all the users agree on the labor-intensiveness of the tool for extracting a single neuron, highlighting the need of an automatic system to perform the task. Finally, since ManSegTool is open-source, it can be widely used as a tool to extract and represent any kind of datasets.

Although the tool was tested on neurons in their own arrangement within the brain, it can be easily used to segment other complex objects (i.e., vasculature, glia cells, porous scaffolds) imaged using different techniques (e.g., two photon microscopy, microCT). Moreover, due to the characteristics of ManSegTool's outputs, the three-dimensional neurons extracted with ManSegTool could also be useful for further studies, such as for simulating the neuron electrophysiological behavior based on empirical microscopic data.

In conclusion, ManSegTool is proposed here as a robust and simple tool to segment complex structures, where automatic or semi-automatic tools and algorithms still fail. The tool could be of interest to researchers involved in developing single-neuron segmentation algorithms. Researchers can test their own results comparing those obtained with ManSegTool in terms of accuracy, precision and time.

## Author contributions

CM, AC, NV, and AA designed the research; CM and AC performed the research; CM, AC, and NV analyzed the data; CM and AA wrote the paper. All authors read and approved the final manuscript.

### Conflict of interest statement

The authors declare that the research was conducted in the absence of any commercial or financial relationships that could be construed as a potential conflict of interest. The reviewer JvP and handling Editor declared their shared affiliation, and the handling Editor states that the process nevertheless met the standards of a fair and objective review.
